# Mitochondrial genome evolution of the hemiparasitic plant *Taxillus*: gene loss, selective constraints, and structural variation

**DOI:** 10.3389/fpls.2026.1898028

**Published:** 2026-07-22

**Authors:** Mengjin Tan, LingYun Wan, Wenjing Liang, Can Chen, Guiyu Tan, Ya Qin, Yuqiong Li, Lisha Song, Haixia Yu, Cuihong Yang, Lingjian Gui, Shugen Wei

**Affiliations:** 1 Guangxi Key Laboratory of High-Quality Formation and Utilization of Dao-di Herbs, Guangxi Botanical Garden of Medicinal Plants, Nanning, China; 2 National Center for Traditional Chinese Medicine (TCM) Inheritance and Innovation, Guangxi Botanical Garden of Medicinal Plants, Nanning, China; 3 Guangxi Key Laboratory for the Production of Traditional Chinese Medicinal Materials with Food and Medicine Homology, Guangxi Botanical Garden of Medicinal Plants, Nanning, China

**Keywords:** gene loss, hemiparasitic plants, mitochondrial genome, parasitic plants, *Taxillus chinensis*

## Abstract

**Background:**

Parasitic and hemiparasitic plants exhibit highly variable mitochondrial genome structures and gene content, but the evolutionary processes underlying this variation remain incompletely understood. This study aimed to characterize the structural features, gene composition, and evolutionary characteristics of the complete mitochondrial genome of *Taxillus chinensis.*

**Results:**

The mitochondrial genome of *T. chinensis* exhibits a complex multipartite structure composed of multiple linear and circular DNA molecules. Fragmented synteny and heterogeneous genomic organization indicate substantial structural complexity. The genome contains abundant dispersed and palindromic repeats, including long repeat units, contributing to the complex repetitive landscape of the mitogenome. The mitogenome encodes 27 protein-coding genes and three rRNA genes, but only three tRNA genes, indicating substantial reduction of mitochondrial tRNA genes. All *rpl* genes are absent, indicating a substantial reduction of translation-related components. Homology searches against the nuclear genome and mitochondrial contigs identified only fragmented rpl-related sequences, which likely represent nonfunctional pseudogene remnants rather than intact functional genes. Ka/Ks analyses show that most mitochondrial protein-coding genes remain under strong purifying selection, although evolutionary rate heterogeneity is observed among functional categories.

**Conclusion:**

The mitochondrial genome of *T. chinensis* exhibits pronounced structural complexity and extensive loss of translation-related genes, while most retained mitochondrial protein-coding genes remain under strong purifying selection. These findings provide new insights into the structural evolution, functional reduction, and evolutionary constraints of mitochondrial genomes in hemiparasitic plants.

## Introduction

*Taxillus chinensis* is a hemiparasitic shrub in the order Santalales, widely distributed in southern China and Southeast Asia. It is an important medicinal plant in traditional Chinese medicine. The pharmacological properties of *Taxillus* species have been partially validated by modern studies, and their extracts have been reported to exhibit anti-rheumatic, liver- and kidney-tonifying, antihypertensive, and anti-hemorrhagic effects during pregnancy (Chinese Pharmacopoeia Commission, 2015; Liu et al., 2019b). The genus *Taxillus* exhibits a broad host range, parasitizing diverse woody plants across multiple families, including Rosaceae, Sapindaceae, Fabaceae, and Theaceae, with *Morus*-associated individuals traditionally considered to have higher medicinal value (Wu and Raven, 1988). *Taxillus* attaches to host plants via haustoria that penetrate the xylem and/or phloem, forming a physiological bridge for the efficient acquisition of water, minerals, and organic assimilates, thereby exhibiting a typical hemiparasitic lifestyle (Wakatake et al., 2020).

Despite its ecological and medicinal importance, genomic resources for *T. chinensis* remain limited, particularly regarding organellar genomes, which have not been systematically investigated. Recently, a chromosome-level nuclear genome assembly revealed significant divergence of orthologous gene clusters between *T. chinensis* and holoparasitic plants such as Cuscuta, with strong enrichment in chloroplast-related functional categories, highlighting functional differentiation between hemiparasitic and holoparasitic lifestyles (Fu et al., 2022). In addition, the complete chloroplast genome of *T. chinensis* (~121 kb) has been characterized, exhibiting the typical quadripartite structure of angiosperms and a close phylogenetic relationship with *Taxillus sutchuenensis* (Liu et al., 2019a). Comparative analyses of chloroplast genomes within Loranthaceae further indicate widespread gene loss and functional degradation among parasitic lineages, particularly extensive loss of the *ndh* gene family, as well as reductions in *rpl, rps*, and tRNA genes, reflecting relaxed selective constraints associated with the parasitic lifestyle. However, current studies on the genus *Taxillus* remain largely descriptive and are based on limited sampling, and comprehensive intraspecific comparative analyses are still lacking, which hampers a deeper understanding of its evolutionary diversity (Su et al., 2021).

Parasitic plants are widely regarded as a highly specialized evolutionary group characterized by genome reduction, gene loss, pseudogenization, and elevated nucleotide substitution rates in their organellar genomes. Plastid genomes in holoparasitic plants exhibit relatively convergent patterns of degradation (Wicke and Naumann, 2018), whereas mitochondrial genomes show pronounced heterogeneity and strong lineage dependence (Skippington et al., 2015b, 2017; Sanchez-Puerta et al., 2023). In holoparasitic plants, mitochondrial genome remodeling is particularly extreme. For example, *Rhopalocnemis phalloides* possesses a highly fragmented mitogenome composed of multiple minicircular chromosomes, accompanied by strong structural plasticity and regional sequence divergence (Yu et al., 2022). In *Cuscuta*, mitochondrial genomes show limited gene loss and lack clear evidence of extensive horizontal gene transfer, yet exhibit notable structural changes, including fragmentation of the *ccmFC* gene and trans-splicing of group II introns (Anderson et al., 2021). These features highlight the extreme structural plasticity of parasitic plant mitogenomes.

In contrast, hemiparasitic plants are generally considered to retain relatively conserved mitochondrial gene content, although structural rearrangements are still frequently observed. In *Cymbaria*, repeat-mediated homologous recombination drives extensive genome rearrangements and generates multipartite or multi-conformational mitochondrial genome structures, while overall gene content remains largely comparable to that of autotrophic angiosperms (Ma et al., 2025). However, increasing evidence suggests that mitochondrial genome evolution in hemiparasites is not uniformly conservative. In the genus *Viscum*, pronounced gene degradation and accelerated sequence evolution have been reported. *Viscum scurruloideum* has lost all complex I-related *nad* genes, accompanied by markedly elevated substitution rates (Skippington et al., 2015a). In *Viscum album*, although several core respiratory genes were initially reported as absent, subsequent re-annotation using more sensitive alignment strategies revealed that some of these genes can still be detected as highly divergent homologs, suggesting that apparent gene loss may partly reflect extreme sequence divergence rather than true absence (Skippington et al., 2017). These findings, together with evidence from other parasitic lineages, suggest that mitochondrial gene degradation in hemiparasitic plants may follow a gradual and lineage-dependent trajectory rather than a binary loss pattern.

Despite these advances, systematic investigations of mitochondrial genome evolution in hemiparasitic plants remain limited, particularly regarding structural organization and the evolution of translation-related genes. In addition, comparative analyses of selective constraints among different functional gene categories remain scarce. In this study, we assembled and annotated the mitochondrial genome of *T. chinensis* and characterized its genome structure, gene content, repeat composition, and syntenic relationships, with particular emphasis on the evolution of translation-related components, including mitochondrial tRNAs and ribosomal protein genes. We further investigated whether nuclear-encoded remnants of missing mitochondrial *rpl* genes were present and conducted Ka/Ks analyses to evaluate selective constraints acting on mitochondrial genes in parasitic and autotrophic angiosperms. Our results reveal a highly complex multipartite mitochondrial genome organization and substantial reduction of translation-related genes in *T. chinensis*, providing new insights into mitochondrial genome evolution and functional reduction in hemiparasitic plants.

## Mitochondrial genome characteristics and gene composition of *Taxillus chinensis*

**Figure 1:**
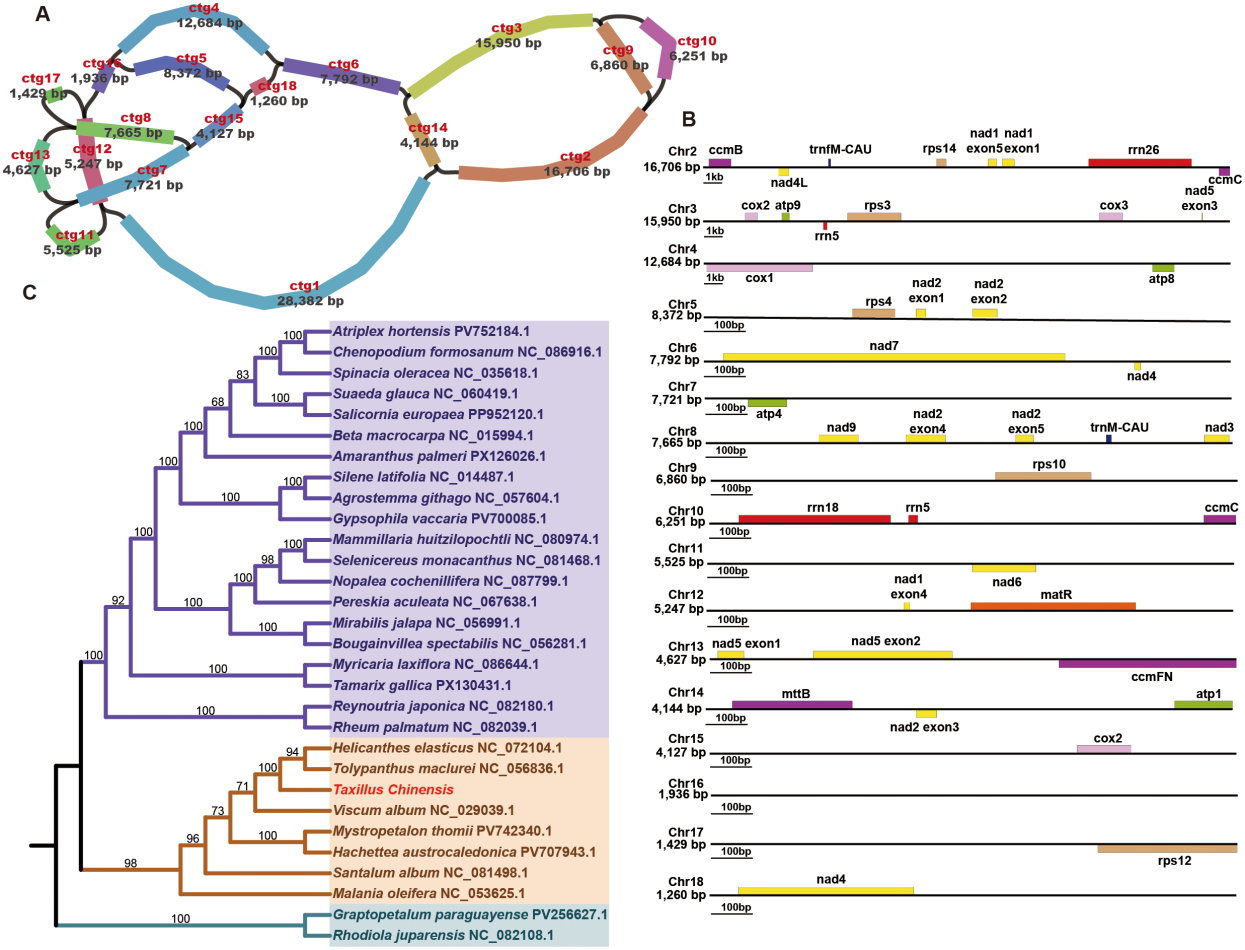
Mitochondrial genome assembly, structure, and repeat sequence analysis. **(A)** Schematic representation of the initial assembly results of the mitochondrial genome. **(B)** Map of the mitochondrial genome. **(C)** Phylogenetic tree of 30 angiosperm species based on the concatenated DNA sequences of 19 conserved mitochondrial protein-coding genes. Two species from Saxifragales were used as outgroups. The topology was largely consistent with the latest APG classification system, and *T. chinensis* was placed within Santalales. Bootstrap support values are shown at the nodes.

PacBio HiFi sequencing generated 16.86 Gb of high-quality long reads (973,663 reads, average length 17.31 kb, N50 = 17.10 kb), providing sufficient depth and long-read continuity to support the assembly and structural resolution of the mitochondrial genome (**Supplementary Table 1**). The mitochondrial genome of *T. chinensis* exhibits a complex multibranched graph structure rather than a single circular molecule (**Figure 1A**). It spans 146,678 bp and comprises 18 interconnected linear and circular DNA molecules (**Supplementary Tables 2**, **S3**). The assembly graph shows multiple circular paths intertwined with linear branches, including typical circular substructures (ctg10 and ctg9). A total of 27 unique protein-coding genes (PCGs) were identified, including 22 core and 5 accessory genes (**Figure 1B**), with an overall GC content of 47.49%. Core respiratory genes are retained, including complex I (*nad1–7*, *nad9*), complex III (*cob*), complex IV (*cox1–3*), and ATP synthase (*atp1*, *atp4*, *atp6*, *atp8*, *atp9*). The genome also contains three rRNA genes (one multi-copy), three tRNA genes, and *mttB*. In addition, pseudogenization was detected in *ccmFC* and *matR* (**Supplementary Table 4**). All large ribosomal subunit genes and most tRNA genes have been lost.

## Phylogenetic position of *Taxillus chinensis*

To determine the phylogenetic placement of *T. chinensis*, a maximum likelihood tree was constructed using concatenated sequences of 19 mitochondrial protein-coding genes from 30 representative angiosperm species (**Figure 1C**; **Supplementary Table 5**), with two Saxifragales species used as outgroups. The resulting topology is largely consistent with the APG IV classification system, and most nodes show high bootstrap support (BS = 100). Within Santalales, *T. chinensis* clusters with *Tolypanthus maclurei*, forming a well-supported monophyletic group. This clade is closely related to species of *Viscum* and Misodendraceae, consistent with previously reported phylogenetic relationships within the order.

## Synteny analysis

**Figure 2:**
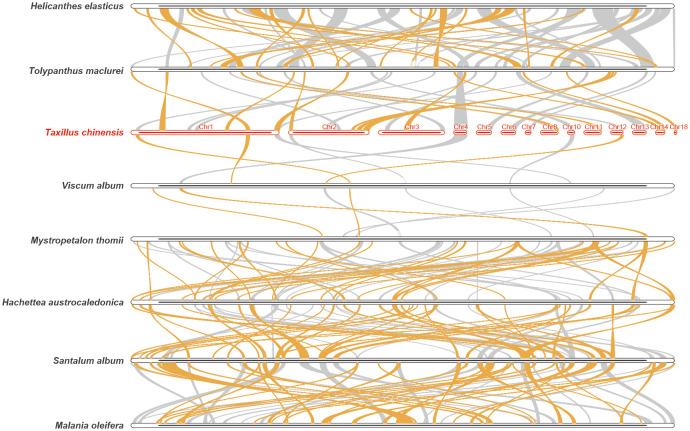
Whole-genome synteny analysis of the *T. chinensis* mitogenome and related species. Gray regions represent homologous syntenic blocks, and orange arcs indicate homologous blocks with opposite orientations. Syntenic blocks shorter than 0.5 kb were excluded.

Synteny analysis between *T. chinensis* and seven closely related Santalales species revealed a generally fragmented pattern of homologous genomic conservation (**Figure 2**, **Supplementary Table 6**). Although several syntenic blocks longer than 500 bp were identified, their distribution among mitochondrial contigs was fragmented compared with related Santalales species such as *Helicanthes elasticus* and *Tolypanthus maclurei.* Overall, conserved gene-containing regions were distributed across multiple mitochondrial segments, corresponding to reduced interspecific syntenic continuity. This pattern reflects a structurally heterogeneous organization of the *T. chinensis* mitogenome relative to other Santalales species.

## Repeat sequence architecture

**Figure 3:**
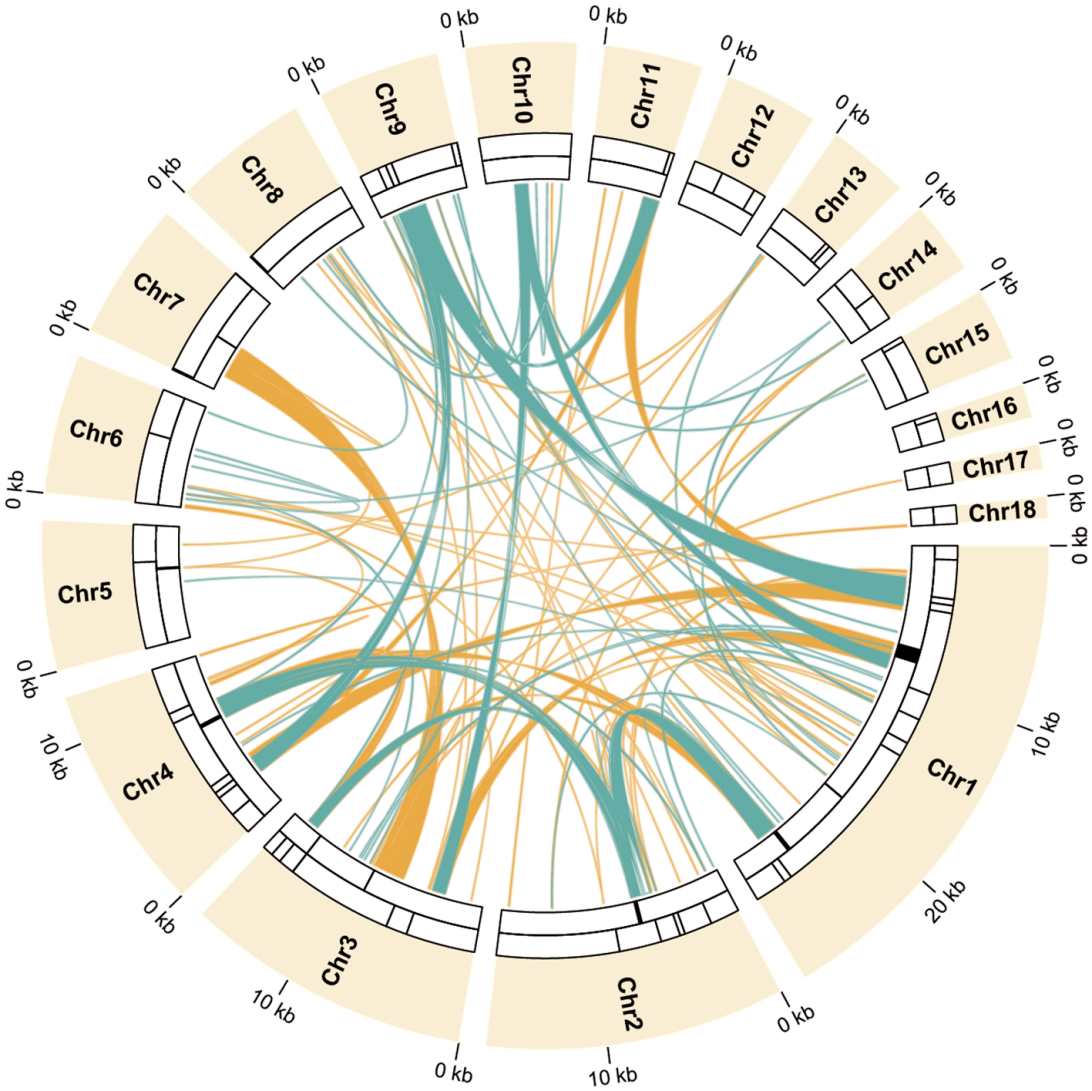
Identification of repeat sequences. Colored links in the innermost circle represent pairs of dispersed repeats, with green and orange lines indicating palindromic and forward repeats, respectively. Black bars in the second circle denote tandem repeats, while those in the outermost circle indicate SSRs.

Three categories of repeat sequences were identified in the mitochondrial genome of *T. chinensis* (**Figure 3**; **Supplementary Tables S7**-**S9**), including simple sequence repeats (SSRs), tandem repeats, and dispersed repeats. A total of 44 SSRs were detected, showing an uneven distribution across chromosomes. Chr 1 contained the highest number of SSRs (10), whereas no SSRs were detected in Chr 10, Chr 17, or Chr 18. Among SSR types, dinucleotide repeats were the most abundant (38.64%), while mononucleotide repeats were not detected. This heterogeneous distribution indicates that repetitive elements are not randomly distributed across the mitochondrial genome. A total of 16 tandem repeats were identified, ranging from 6–475 bp in length, with sequence identities exceeding 67%. Their chromosomal distribution was largely consistent with that of SSRs, with enrichment in Chr1 and absence in several smaller chromosomes, further supporting the heterogeneous distribution of repetitive elements across the genome.

Dispersed repeats represented the predominant repeat category, with 196 repeat pairs (≥30 bp) identified, including 97 palindromic repeats. The abundance of dispersed repeats indicates that they constitute a major component of the repetitive landscape of the *T. chinensis* mitochondrial genome. Several long repeat sequences were detected, with the longest repeat unit reaching 2,445 bp. These large repeats were distributed across different mitochondrial contigs and contributed to the complex repeat architecture of the genome. Further characterization of the largest repeat unit (R1, 2,445 bp) revealed variable genomic locations among repeat copies. One copy was located within the region containing the core gene *atp4* on Chr7, whereas other copies were primarily located in intergenic regions. This distribution pattern suggests that repetitive sequences are associated with diverse genomic contexts in the *T. chinensis* mitochondrial genome.

## Loss of translation-related genes

**Figure 4:**
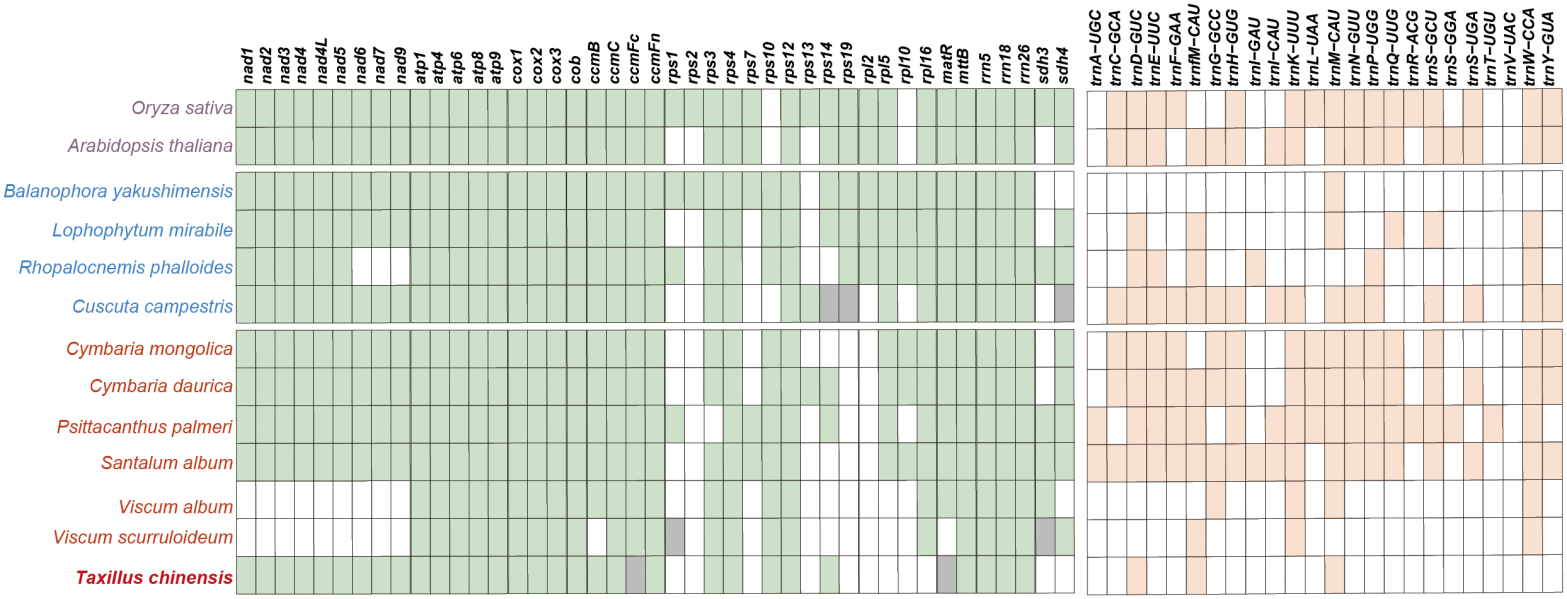
Distribution and annotation of protein-coding genes and tRNA genes in the mitochondrial genomes of hemiparasitic, holoparasitic, and autotrophic plants. Green and orange indicate intact genes, grey indicates pseudogenes, and white indicates gene absence. Blue, red, and purple labels denote parasitic, hemiparasitic, and autotrophic species, respectively. The same set of species was used for both protein-coding gene and tRNA gene comparisons to ensure consistency between panels.

Compared with autotrophic plants, parasitic plants exhibit a substantial loss of mitochondrial protein-coding genes (**Figure 4**). In *T. chinensis*, all ribosomal large subunit protein genes (*rpl*) were lost, whereas only a limited subset of ribosomal small subunit protein genes (*rps3, rps4, rps10, rps12, and rps14*) was retained. Furthermore, only three mitochondrial tRNA genes were identified in the mitogenome (*trnD-GUC*, *trnfM-CAU*, and *trnM-CAU*), indicating a markedly reduced intrinsic translational capacity of the mitogenome (**Supplementary Table 4**). Several sequence fragments showing similarity to mitochondrial *rpl* genes were identified in the nuclear genome of *T. chinensis* based on TBLASTN searches using RPL proteins from *Arabidopsis thaliana* as queries. However, these sequences were highly fragmented, showed only low to moderate sequence similarity, and lacked intact open reading frames and conserved protein domains, suggesting that they likely represent degenerated pseudogene remnants rather than functional nuclear-encoded *rpl* homologs (**Supplementary Table 10**). In addition, homology searches were performed against both the assembled mitochondrial contigs of *T. chinensis*. No complete *rpl* gene sequences were detected in the dataset, further supporting the absence of functional mitochondrial *rpl* genes (**Supplementary Table 11**).

Comparative analyses of mitochondrial tRNA gene composition among parasitic and autotrophic plants further revealed a substantial reduction of mitochondrial tRNA genes in *T. chinensis.* Only three mitochondrial tRNA types, *trnD-GUC*, *trnfM-CAU*, and t*rnM-CAU*, were identified in the mitogenome (**Supplementary Table 3**). These annotations were further confirmed by BLASTn searches using tRNA genes from *Arabidopsis thaliana*, which confirmed the presence of the same three tRNA genes in the assembled mitochondrial contigs with high sequence identity (>93%) and full-length alignments (**Supplementary Table 12**). Closely related hemiparasites such as *Viscum album* and *Viscum scurruloideum* also exhibit extensive mitochondrial tRNA gene loss. Interestingly, however, some hemiparasites, such as *Cymbaria daurica* and *Cymbaria mongolica*, as well as the holoparasite *Cuscuta campestris*, retain nearly complete mitochondrial tRNA gene sets comparable to those of autotrophic plants (**Figure 4**). Autotrophic angiosperms, such as *Arabidopsis thaliana* and *Oryza sativa*, typically retain approximately 20–25 mitochondrial tRNA genes (Warren and Sloan, 2020).

Notably, the retained tRNA genes in *T. chinensis* include *trnfM-CAU* and t*rnD-GUC*, which are among the most conserved mitochondrial tRNA types in plants and are expected to be associated with core translational functions. The *trnM-CAU* sequence showed high similarity to chloroplast-derived sequences based on comparative analysis with the plastid genome assembled in this study (**Supplementary Tables 13**, **14**), suggesting a potential history of intracellular gene transfer. Taken together, these results indicate that *T. chinensis* possesses a highly reduced mitochondrial tRNA repertoire, with the remaining tRNA-related sequences likely reflecting a combination of conserved functional retention and plastid-derived sequence transfer.

## Variation in selective pressure on mitochondrial gene evolution associated with parasitic lifestyle

**Figure 5:**
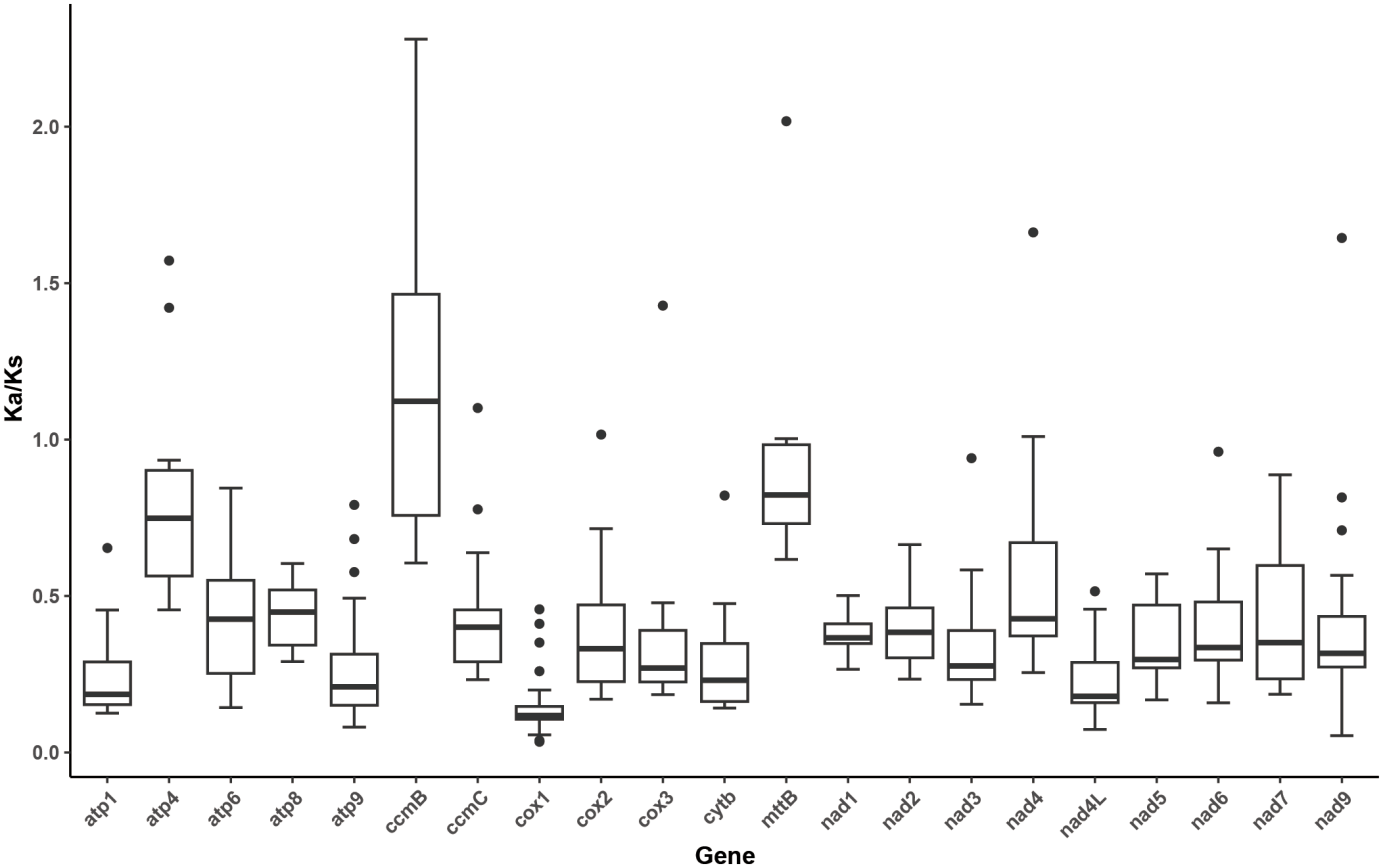
Ka/Ks ratios of mitochondrial genes in eight angiosperms.

To evaluate the evolutionary rates and selective pressures acting on mitochondrial PCGs, Ka/Ks analyses were conducted across 21 mitochondrial genes from eight angiosperm species (**Figure 5**). Overall, most genes exhibited median Ka/Ks values substantially below 1, generally ranging from ~0.1–0.6, indicating pervasive purifying selection and strong evolutionary conservation of the mitochondrial genome.

Marked heterogeneity was observed among genes. Core respiratory chain genes, including *cox1*, *cox2*, and *atp9*, consistently showed low Ka/Ks values with narrow distributions and limited variation, suggesting strong functional constraints across species pairs. In contrast, genes such as *atp4*, *ccmB*, and *nad7* displayed higher median Ka/Ks values and broader distributions, with occasional values exceeding 1 in specific comparisons, indicating relaxed selective constraints and/or lineage-specific accelerated evolution. In parasite- and hemiparasite-associated lineages, several genes exhibited moderately elevated Ka/Ks values relative to autotrophic species comparisons. However, this pattern was primarily reflected in shifts in distribution and increased variability rather than a consistent genome-wide trend, suggesting that the impact of parasitic lifestyle on mitochondrial gene evolution is gene- and lineage-specific.

Although Ka/Ks values exceeding 1 were detected in a few cases (*ccmB* and *atp4*), these signals were sporadic and not consistently recovered across comparisons, and therefore likely reflect relaxed selection or localized sequence divergence rather than strong evidence of pervasive positive selection. Overall, mitochondrial gene evolution in these angiosperms is dominated by purifying selection, while functional gene categories exhibit distinct evolutionary constraints. Parasitic lineages may experience partial relaxation of selective pressure in specific genes, although this effect is heterogeneous and context-dependent.

## Discussion

Mitochondrial genome studies in hemiparasitic plants remain limited, restricting our understanding of their structural evolution and functional adaptation. In this study, we assembled the complete mitochondrial genome of *T. chinensis* using PacBio long-read sequencing and systematically characterized its genome architecture, gene content, repeat composition, phylogenetic position, and evolutionary patterns. The relatively uniform sequencing depth supports the reliability of the assembly and provides a valuable resource for future studies of parasitic plant mitochondrial evolution.

The *T. chinensis* mitogenome exhibits substantial structural dynamism, including multipartite organization, fragmented synteny, and heterogeneous genomic organization. Plant mitochondrial genomes are well known for their structural plasticity (Arrieta-Montiel and Mackenzie, 2011; Christensen, 2013). In *T. chinensis*, abundant dispersed and palindromic repeats were identified, and several rearrangement breakpoints were associated with repeat-rich regions, suggesting that repetitive elements may contribute to genome restructuring. Compared with most angiosperm mitochondrial genomes, the repeat landscape of *T. chinensis* appears more complex, indicating increased structural complexity during mitochondrial genome evolution.

Concurrently, the *T. chinensis* mitogenome exhibits pronounced reduction of translation-related components. All mitochondrial *rpl* genes were lost, whereas only three tRNA genes (*trnD-GUC*, *trnfM-CAU*, and *trnM-CAU*) were retained. Similar reductions have been reported in several parasitic plants, particularly in highly reduced mitochondrial genomes (Skippington et al., 2015b; Sanchez-Puerta et al., 2019). However, such losses are not universal among parasitic angiosperms. For example, the hemiparasite *Santalum album* and the holoparasite *Cuscuta campestris* (Anderson et al., 2021) retain most of their mitochondrial tRNA genes despite their parasitic lifestyles. This contrast suggests that extensive loss of translational genes is not an inevitable consequence of parasitism, but rather reflects lineage-specific evolutionary trajectories. The marked reduction of tRNA and ribosomal protein genes in *T. chinensis* may be associated with relaxed selective constraints following the transition to a parasitic lifestyle (Bromham et al., 2013; Wicke et al., 2016). Under reduced dependence on autonomous organellar gene expression, genes that can be functionally replaced by nuclear-encoded products or imported molecules may become dispensable and subsequently be lost during genome evolution. Similar patterns of gene loss driven by relaxed selection have been proposed for both mitochondrial and plastid genomes of parasitic plants (Wicke et al., 2013; Wicke and Naumann, 2018). Consequently, organellar genome evolution in parasitic lineages appears to be characterized by progressive genomic simplification, although the extent of reduction varies substantially among taxa, indicating distinct adaptive evolutionary pathways.In addition, one retained tRNA in *T. chinensis* appears to be plastid-derived, supporting the long-recognized role of intracellular gene transfer in shaping plant mitochondrial genomes (Kubo and Newton, 2008). Under extensive mitochondrial gene loss, such transferred tRNA sequences may represent potential contributions to the remaining mitochondrial translational system. Together with the absence of *rpl* genes, these observations suggest progressive simplification of the mitochondrial translational system and increasing dependence on nuclear-encoded components. Because plant mitochondria already rely heavily on nuclear-encoded proteins for their maintenance and function (Adams et al., 2002), large-scale loss of translational genes may further promote functional outsourcing to the nucleus, potentially involving enhanced tRNA import and protein targeting mechanisms (Salinas-Giegé et al., 2015).

Despite extensive reduction of translation-related genes, core oxidative phosphorylation genes remain highly conserved and are subject to strong purifying selection. Most mitochondrial protein-coding genes exhibited Ka/Ks values substantially lower than 1, indicating overall functional conservation. However, selective constraints varied among functional categories. Core respiratory genes such as *cox1*, *cox2*, and *atp9* showed consistently low evolutionary rates, whereas several auxiliary genes displayed relatively relaxed selective constraints. Similar asymmetric evolutionary patterns have been observed in other parasitic plants (Bromham et al., 2013; Xu et al., 2022; Cai et al., 2026), suggesting that parasitic adaptation preferentially affects auxiliary mitochondrial functions while preserving essential energy metabolism.

Taken together, our results support an evolutionary scenario in which parasitic adaptation promotes both genome structural diversification and functional streamlining in plant mitochondrial genomes. Although essential respiratory functions remain evolutionarily conserved, translational components appear to undergo progressive reduction, reflecting asymmetric evolutionary constraints during adaptation to parasitic lifestyles.

## Methods

### Plant material and sequencing

Fresh young leaves of *T. chinensis* used in this study were collected from the medicinal plant cultivation and research base of the Guangxi Botanical Garden of Medicinal Plants, Nanning, Guangxi, China. The species was identified by Professor Shugen Wei. Collected samples were immediately frozen in liquid nitrogen and subsequently subjected to PacBio HiFi sequencing.

### Mitogenome sequencing and assembly

The mitochondrial genome was assembled from PacBio HiFi reads using OATK (Zhou et al., 2025) (“syncasm -k 1001 -c 30”), producing a GFA-format assembly graph. Contigs were compiled into a local BLAST database using makeblastdb. Mitochondrial gene sequences from *Tolypanthus maclurei* (NC_056836.1) were used as queries in BLASTn (−evalue 1e−5, −outfmt 6, −max_hsps 10, −word_size 7, −task blastn-short) to identify mitochondrial contigs. The assembly graph was visualized in Bandage (v0.8.1) (Wick et al., 2015), and candidate mitochondrial contigs were selected based on BLASTn hits and extracted to generate the draft mitochondrial genome.

### Mitogenome annotation

The mitochondrial genome was annotated using PMGA (Li et al., 2025) (http://www.1kmpg.cn/pmga/) with a reference set of 319 angiosperm mitochondrial genomes. tRNA genes were predicted using tRNAscan-SE (v2.0.11) (Lowe and Eddy, 1997), and rRNA genes were identified by BLASTN (v2.13.0) (Chen et al., 2015). Annotations were manually curated and corrected in Apollo (v1.11.8) (Lewis et al., 2002). The mitochondrial genome map was generated using OGDRAW (Greiner et al., 2019).

### Phylogenetic analysis

Shared genes were extracted using PhyloSuite (v1.1.16) (Zhang et al., 2020). Multiple sequence alignment was performed using MAFFT (v7.505) (Katoh and Standley, 2013). A maximum likelihood (ML) phylogenetic tree was constructed using IQ-TREE (v1.6.12) (Nguyen et al., 2015) with parameters “--alrt 1000 -B 1000”.The resulting phylogenetic tree was visualized and annotated using iTOL (v6) (Letunic and Bork, 2019).

### Repetitive sequence identification

SSRs, tandem repeats, and dispersed repeats were identified using MISA (v2.1) (Beier et al., 2017), TRF (v4.09) (Benson, 1999), and REPuter (Wynn and Christensen, 2019). Visualization was performed using Microsoft Excel (2021) and Circos (v0.69.9) (Zhang et al., 2013).

### Synteny analysis

Syntenic blocks were identified using BLASTn with the parameters “-evalue 1e-5, -word_size 9, -gapopen 5, -gapextend 2, -reward 2, -penalty -3”. Only syntenic blocks longer than 500 bp were retained for subsequent analyses. Pairwise synteny analysis was performed using MCScanX (Wang et al., 2012) based on BLASTn results, and multiple synteny plots were generated to visualize conserved collinear blocks.

### Chloroplast genome assembly, annotation, and homologous fragment analysis

The chloroplast genome was assembled using OATK (Zhou et al., 2025) based on PacBio HiFi sequencing data. Genome annotation was performed using CPGAVAS2 (Shi et al., 2019), and the annotation results were further manually inspected and corrected using CPGView (Liu et al., 2023). Homologous fragments were identified using BLASTN (v2.13.0) (Chen et al., 2015), and the results were visualized using the Circos package (v0.69.9) (Zhang et al., 2013).

### Ka/Ks analysis

PCGs from eight angiosperm species were extracted using PhyloSuite (v1.1.16). Codon-aware alignments were performed using MACSE (v5.3) (Ranwez et al., 2018) to ensure accurate reading frame alignment. Sequences containing internal stop codons or ambiguous regions were removed prior to analysis. Pairwise nonsynonymous (Ka) and synonymous (Ks) substitution rates were calculated using KaKs_Calculator3.0 (Zhang, 2022) under the GY model. Ka/Ks ratios were used to infer selective pressure acting on mitochondrial genes.

## Data Availability

The mitochondrial genome sequences of *Taxillus chinensis* have been deposited in the NCBI database under accession numbers PZ467582–PZ467599.

## Supplementary material

Supplementary Table 1 Summary statistics of PacBio HiFi sequencing data for *Taxillus chinensis* mitochondrial genome project.

Supplementary Table 2 Contig length and sequencing depth of the mitochondrial genome assembly.

Supplementary Table 3 Genomic features of the *Taxillus chinensis* mitochondrial genome.

Supplementary Table 4 Protein-coding genes identified in the *Taxillus chinensis* mitochondrial genome.

Supplementary Table 5 Taxa and GenBank accession numbers used in phylogenetic analysis.

Supplementary Table 6 Sequence alignment statistics for synteny analysis.

Supplementary Table 7 Dispersed repeat sequences in the *Taxillus chinensis* mitochondrial genome.

Supplementary Table 8 SSRs in the mitochondrial genome of *Taxillus chinensis*.

Supplementary Table 9 Tandem repeat sequences in the mitochondrial genome of *Taxillus chinensis*.

Supplementary Table 10 Putative nuclear homologs of mitochondrial *rpl* genes identified by TBLASTN searches.

Supplementary Table 11 TBLASTN results of mitochondrial RPL proteins against the mitochondrial genome of *Taxillus chinensis*.

Supplementary Table 12 Evolutionary status of mitochondrial tRNA genes in *Taxillus chinensis*.

Supplementary Table 13 Structural organization of the chloroplast genome of *Taxillus chinensis*.

Supplementary Table 14 Homologous DNA fragments identified in the *Taxillus chinensis* mitochondrial genome.
